# Psychosocial Health Problems Associated with Increased HIV Risk Behavior among Men Who Have Sex with Men in Nepal: A Cross-Sectional Survey

**DOI:** 10.1371/journal.pone.0058099

**Published:** 2013-03-13

**Authors:** Keshab Deuba, Anna Mia Ekström, Rachana Shrestha, George Ionita, Laxmi Bhatta, Deepak Kumar Karki

**Affiliations:** 1 Department of Public Health Sciences, Karolinska Institutet, Stockholm, Sweden; 2 Division of Global Health (IHCAR), Department of Public Health Sciences, Karolinska Institutet, Stockholm, Sweden; 3 Department of Infectious Diseases, Huddinge, Karolinska University Hospital, Stockholm, Sweden; 4 Nobel College, Pokhara University, Kathmandu, Nepal; 5 United Nations Development Programme (UNDP), Kathmandu, Nepal; 6 National Centre for AIDS and STD Control, Kathmandu, Nepal; University of Washington, United States of America

## Abstract

**Background:**

Men who have sex with men (MSM) are marginalized, hidden, underserved and at high risk for HIV in Nepal. We examined the association between MSM sub-populations, psychosocial health problems and support, access to prevention and non-use of condoms.

**Methods:**

Between September-November of 2010, a cross-sectional survey on HIV-related risk behavior was performed across Nepal through snowball sampling facilitated by non-governmental organizations, recruiting 339 MSM, age 15 or older. The primary outcomes were: (a) non-use of condoms at least once in last three anal sex encounters with men and (b) non-use of condoms with women in the last encounter. The secondary outcome was participation in HIV prevention interventions in the past year.

**Results:**

Among the 339 MSM interviewed, 78% did not use condoms at their last anal sex with another man, 35% did not use condoms in their last sex with a woman, 70% had experienced violence in the last 12 months, 61% were experiencing depression and 47% had thought of committing suicide. After adjustment for age, religion, marital status, and MSM subpopulations (bisexual, *ta*, *meti*, gay), non-use of condoms at last anal sex with a man was significantly associated with non-participation in HIV interventions, experience of physical and sexual violence, depression, repeated suicidal thoughts, small social support network and being dissatisfied with social support. Depression was marginally associated with non-use of condoms with women. The findings suggest that among MSM who reported non-use of condoms at last anal sex, the *ta subgroup* and those lacking family acceptance were the least likely to have participated in any preventive interventions.

**Conclusions:**

MSM in Nepal have a prevalence of psychosocial health problems in turn associated with high risk behavior for HIV. Future HIV prevention efforts targeting MSM in Nepal should cover all MSM subpopulations and prioritize psychosocial health interventions.

## Introduction

Male-to-male sex contributes substantially to the HIV epidemic in Asia [1]. In Nepal, the first case of HIV infection was reported in 1988 [2]. Since then, the HIV epidemic has evolved from a low to a concentrated epidemic among identified key vulnerable populations at higher risk of becoming infected with HIV: men who have sex with men (MSM), people who inject drugs, female sex workers, and male labor migrants, particularly those migrating between Nepal and high HIV prevalence areas in India. A decade (1996–2006) of armed conflict between government forces and rebel forces, an unstable political situation, and an obsolete health policy (from 1991) has undermined an already weak health care system, resulting in poor service delivery and access to prevention and care [3]–[4]. In the present political vacuum in Nepal, the lack of government initiatives to reduce human rights violations towards vulnerable populations such as MSM and transgender individuals has led to further deterioration of their health and living conditions, including increased risk of sexual violence and discrimination. In Nepal, the risk of acquiring HIV infection among MSM is 9.2 times higher than for the adult general population [5] and the prevalence of HIV is relatively stable, from 3.9% in 2004 [6] to 3.8% in 2009 [7], though it increased to 6.3% in 2012 [8]. Given the high rate of bisexual relationships [9], [10], prevention of HIV infections among MSM in Nepal would also protect women and children. However, the generalized discomfort around, and existing stigma towards, male-male sex in Nepal, results in discrimination, violence and rejection [9]–[11] in turn affecting the effectiveness of existing prevention interventions.

A national HIV surveillance system was established in the early nineties to monitor the dynamics of the epidemic and to inform appropriate interventions. Nepal has a concentrated epidemic with a consistently low estimated HIV prevalence of 0.30% in the general population between the ages of 15–49 in 2012 [12]. Thus, HIV prevention is primarily targeted to key vulnerable populations at higher risk to HIV and population-based studies of the HIV prevalence in MSM began in 2004. In 2007, 69% of the US$ 20.3 million allocated for HIV by the Nepalese government was invested in prevention [13]. Among MSM, prevention programs have explicitly focused on traditional interventions such as peer education, condom and lubricant distribution [13]. However, growing evidence suggests that psychosocial health problems such as substance use, partner violence, low social support, isolation, depression, and suicidality act as a syndemic, i.e. interact synergistically to increase HIV related risk behavior among MSM [14]–[17].

A better understanding of how psychosocial syndemic may affect risk behavior among MSM sub-populations has important programmatic implications for future prevention programs. In order to facilitate and inform policy with regards to HIV prevention among MSM, we examined the relationship between various MSM subpopulations, sex roles, other demographic factors, psychosocial health problems, access to preventive interventions, and, non-use of condoms among MSM in Nepal.

## Methods

### Study population

Our study population was men who have sex with other men regardless of how they identified themselves (e.g. gay, bisexual, homosexual, or heterosexual). Participants were eligible for the study if they were at least 15 years of age and reported to have had anal sex with another man in the past 12 months.

### Sampling and recruitment

**Figure 1:**
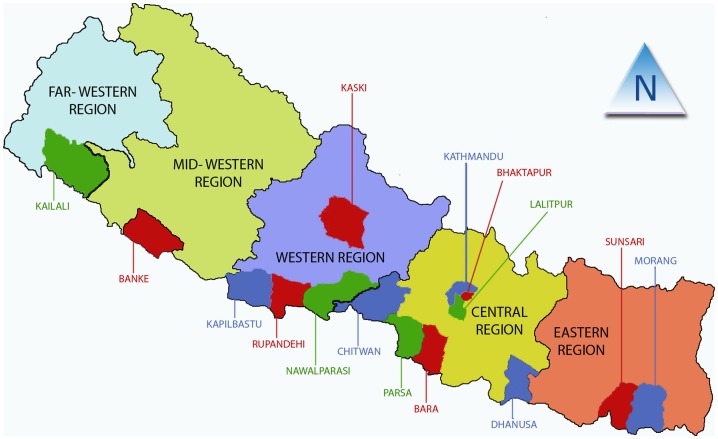
Sampled districts.

Homosexual behavior is widely stigmatized in Nepal [10] because it is considered to be an unnatural behavior and potential study participants are usually hidden in society. Thus, we used outreach workers from nongovernmental organizations (NGOs) working with MSM in 15 different districts of Nepal, to recruit 339 study participants (106 from the central region, 86 from the western region, 54 from the eastern region, 48 from the mid-western region, and 45 from the far-western region) from a wide range of MSM meeting venues between September and November 2010 (Figure 1).

Initially, districts were identified and selected in coordination with the Blue Diamond Society (BDS), an NGO that works with sexual minorities on issues related to sexual health, including HIV, in Nepal. An estimated total number of possible study participants from each district that could be enrolled in the survey were identified in consultation with staff from the BDS. The BDS and its sister organizations of outreach workers in the 15 districts were thereafter informed about the data collection plan and began to recruit possible study participants, so called ‘seeds’, for a face-to-face interview. Finally, a snow-ball sampling technique [18] was used to find harder-to reach MSM by contacting the network of initial ‘seeds’. The response rate was extremely high and none of the invited potential study participants refused to participate in the study. Previous researchers have sometimes used respondent-driven sampling (RDS) to recruit hidden populations, a technique which has also allowed an estimation of selection probabilities [18]. We opted not to use RDS since there was no previous experience of using RDS techniques in this geographically scattered study area that included different districts in Nepal. Also, the overlapping nature of social MSM networks makes it difficult to avoid duplicate enrollment of potential study participants. To avoid or diminish any potential source of selection bias, extensive efforts were made to reach diverse subgroups of MSM in relation to age, occupation, and sex role, from a wide range of venues (e.g. parks, bus stations, temple areas) across a high number of geographical regions throughout the country.

### Study instruments

Before data collection, the semi-structured survey questionnaire was reviewed by BDS staff members and also piloted on six MSM in the Kathmandu valley to test and ensure that the wording of the questions was acceptable and that the response categories were complete. The final study questionnaire and informed consent form were translated into Nepali and back–translated into English to ensure accuracy. Specifically trained public health undergraduate students thereafter conducted face-to-face interviews to collect data on outcomes, demographics and psychosocial variables. Most of the interviews were conducted in MSM outreach or counseling centres but a few interviews were performed in MSM cruising areas (if requested by the study participants).

### Outcome variables

To be comparable with a previous study in a similar cultural context [19], the primary outcome variables indicating increased HIV risk behavior were self-reported non-use of condoms by MSM (a) during anal sex with at least one man in the last three encounters and (b) during anal and or vaginal sex with a woman in the last encounter. A secondary outcome variable (also analyzed as a potential risk factor for the primary outcome) was self-reported participation in at least one HIV prevention intervention in the past year (e.g. having attended MSM outreach/drop-in centers and/or participated in individual counseling, an HIV education session, and or a condom distribution campaign).

### Participant characteristics

Age, religion, education, marital status, sexual orientation, and occupation were recorded. Sexual relationships with women including wives, girlfriends and/or female sex workers in last 12 months were also assessed by asking about female partners with whom the respondent may have had sex, with or without financial transactions for sexual services.

Sexual orientation was based on two dimensions: sexual self-identification and sex role. Sexual self-identification was assessed by asking ‘Which of the following MSM subpopulation categories describes you the best (only one answer allowed): bisexual, *ta*, straight men, homosexual/gay, or *meti*?’ Sex role was based on the answer to ‘What type of sex role have you had: anal insertive, anal receptive, both insertive and receptive or oral only?’

### Definition of sexual identification terms

The description of sexual identification terms: Meti: Effeminate men attracted to the same-sex, who sometimes cross-dress and have a receptive role during anal sex; Ta: Masculine appearing men and sexual partners of meti; Bisexual: Men who are sexually attracted towards both men and women; and Homosexual/gay: This is a broader term representing sexual orientation (attraction to the same sex). In Nepal, some upper and middle class men, especially educated ones, use this term to describe their sexual orientation.

### Psychosocial health problems

#### Depression

Clinically significant depression symptoms were assessed using the Center for Epidemiological Studies Depression Scale (CES-D Scale) [20], a 20-item questionnaire to assess various depressive symptoms experienced in the past week. The responses were scored on a 4-point Likert scale from 0 to 3. The CES-D scale score with a cut-off of 22 or greater was used to define major depression symptoms [14].

#### Violence and victimization experiences

Three forms of violence or verbal abuse in the last 12 months were recorded: use of derogatory words to indicate sexuality e.g., *bhallu* (prostitute) and *chhakka* (infertile man) and verbal threats; physical abuse (slapped, kicked or hit by anyone), and sexual abuse (forced to have non-consensual sex). Victimization experiences were based on physical and sexual abuse or assault by a spouse/sexual partners/relative vs. other than a spouse/sexual partners/relative [21].

#### Suicidality

Assessment of suicidality was based on suicidal ideation (ever considered committing suicide), frequency of suicidal ideation in last 12 months (once/twice, sometimes and always), and ever having planned suicide.

#### Substance use disorders

Two types of substance use disorders (abuse and dependence) were assessed through the Alcohol Use Disorder and Associated Disabilities Interview Schedule IV (AUDADIS-IV)—Diagnostic and Statistical Manual of Mental Disorders, Fourth Edition (DSM-IV) [22], [23]. Substance abuse was assessed regardless of substance dependence [24].

Alcohol and drug (marijuana, cocaine, hallucinogens, inhalants, heroin, sedatives, tranquilizers, pain medications and other stimulants) dependence was defined as a participant having experienced three of the following seven criteria for the particular substance in the last 12 months: tolerance; withdrawal; substance often taken in larger amounts or over a longer period than intended; persistent desire or unsuccessful efforts to cut down or control use; a large amount of time spent on activities to obtain, use, or recover from the substance; important social, occupational, or recreational activities given up or reduced; and continued use despite knowledge of having a persistent or recurrent physical or psychological problem likely to have been caused or exacerbated by the substance [24].

Alcohol and drug abuse was defined as having experienced one or more of the following four behaviors: failure to fulfil major obligations at work, school, or home; recurrent use in situations in which it is physically hazardous; recurrent substance-related legal problems; and continued alcohol and/or drug use despite persistent social or interpersonal problems [24].

### Other socio-behavioral factors

#### Social Support

The Social Support Questionnaire (SSQ) short form was used to assess social support questionnaire number score (SSQN) and social support questionnaire satisfaction score (SSQS) [25]. The SSQN includes 6 questions to elicit the number of supportive persons who can be counted on for different forms of social support. The SSQS was used to assess satisfaction level from available support in a Likert scale that ranges from 1–6: 1 = very dissatisfied, 2 = fairly dissatisfied, 3 = a little dissatisfied, 4 = a little satisfied, 5 = fairly satisfied, and 6 = very satisfied [25]. Higher SSQN and SSQS scores indicate higher level of perceived supportive persons in the social network and satisfaction from available support, respectively, but there is no standard cut-off level that segregates high vs. low SSQS and SSQN. The SSQN and SSQS variables were therefore categorized into quintiles, and the median, respectively, to predict the effect on outcome [26].

#### Family acceptance

A dichotomous measure of a family acceptance was assessed by asking, ‘Is there at least someone in your immediate family that you can talk openly with about your homosexual/bisexual behavior?’

### Statistical analysis

Data was entered using EpiData 3.02 version (www.epidata.dk). Range and consistency checks were done to ensure accuracy, after which data was exported to SPSS 17.0 version for statistical analysis.

Mean, standard deviation (SD), median and interquartile range or ranges were computed for continuous variables and frequencies for categorical variables. Statistically significant associations between independent variables and the outcome variable were computed using first bivariate and then multivariate logistic regression adjusted for age (as a continuous variable), marital status (married vs. single category), religion, and MSM subpopulation (bisexual, *ta*, *meti*, gay). Fisher's exact test was used when one or more of the cells contained values less than or equal to 5. The logistic model fitness was checked by Hosmer-Lemeshow goodness-of-fit test. The confidence interval (CI) was set at 95%, and significance level was set at 0.05.

### Ethical approval

Ethical approval was obtained from Nepal Health Research Council (NHRC), Nepal. Oral informed consent (Appendix S1) was obtained individually from all participants following a detailed description of the study protocol. The consent procedure was well documented and each interviewer signed a statement verifying that he or she had read the informed consent form to provide necessary information and that each study participant had consented to participate in the study. The Nepal Health Research Council was informed that minors would be included in this cross-sectional survey and asked for informed consent. Young people from age 15 years upwards are often included in different surveys conducted in Nepal [27] and informed consent is often sought in the presence of outreach workers from NGOs. No personal identifiers were recorded to ensure confidentiality. Participants were not given any compensation for participating in the study.

## Results

### Participant characteristics

**Figure 2:**
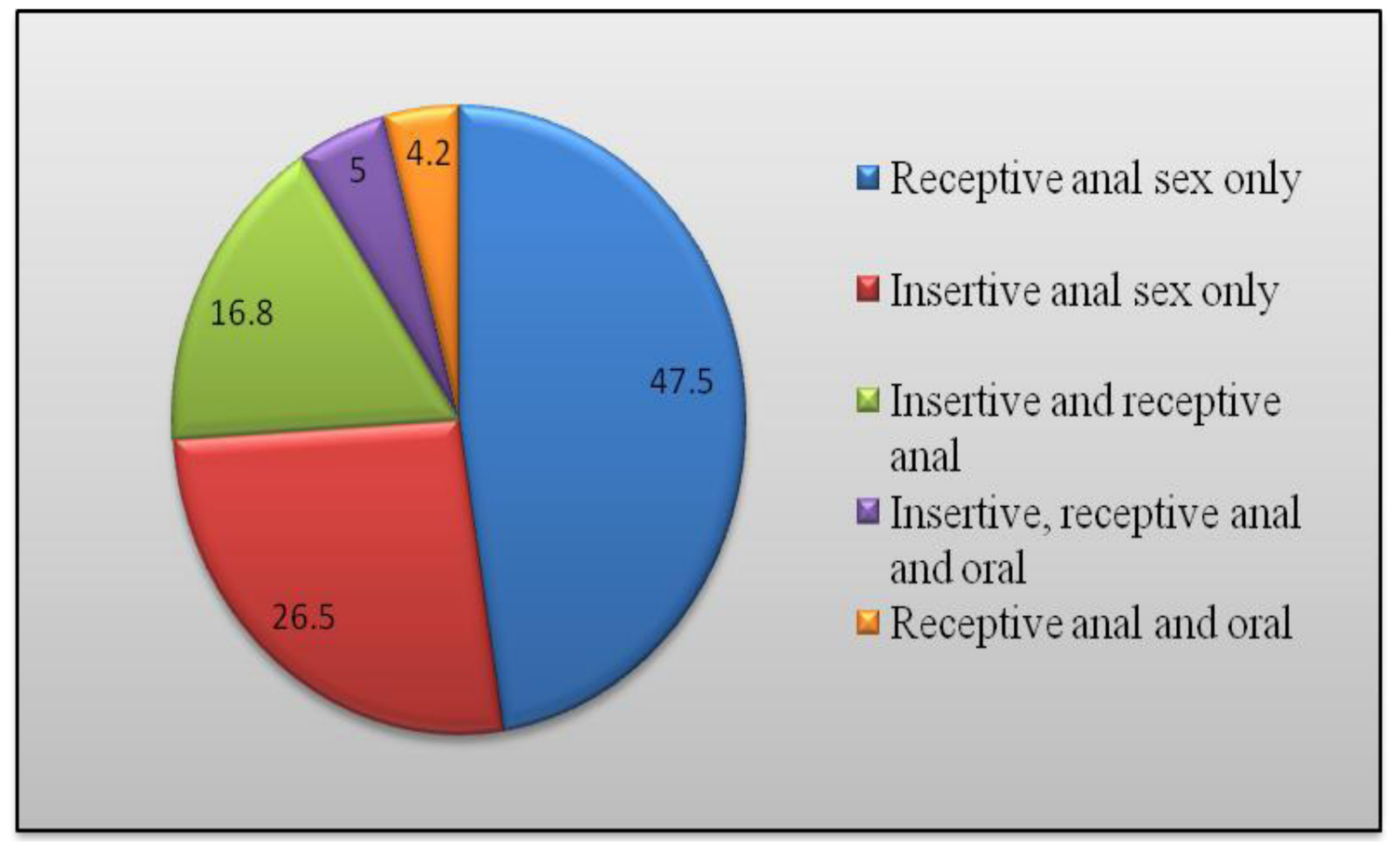
Sex roles in percentages among 339 surveyed MSM in Nepal.

The age of the 339 participants ranged from 15 to 55 years and 57% belonged to the 20–24 and 25–29 age groups. Just over one third of the participants (37%) were married to women, 8% were married to men, and 2% were married to both men and women. More than half (57%) of participants self identified their sexual orientation as *meti* (receptive sex role), followed by homosexuals/gay (13%), bisexual (12%), *ta* (insertive sex role, 9%), and straight (9%). Almost half (48%) reported being a sex worker as their occupation and 86% had participated in any HIV prevention intervention in the past year (Table 1). Almost half (48%) of the total study participants were involved in receptive anal sex, followed by insertive anal only (27%), insertive and receptive anal (17%), insertive, receptive and oral (5%), and receptive and oral (4%) (Figure 2).

**Table 1:** Characteristics of 339 MSM surveyed in Nepal in 2010.

Characteristic	N (%)
Age group	
15–19	34 (10)
20–24	113 (33.3)
25–29	80 (23.6)
30–34	46 (13.6)
35 years and above	66 (19.5)
Religion	
Hindu	288 (85)
Buddhist	25 (7.4)
Christian	16 (4.7)
Muslim	10 (2.9)
Occupation	
Full-time sex worker	119 (35.1)
Part-time sex worker^a^	43 (12.7)
Other occupation	
Unemployed	16 (4.7)
Businessmen	17 (5.0)
Wage laborer/farmer	42 (12.4)
Student	51 (15.0)
Private company staff	90 (26.6)
Civil servant/police/army/driver	4 (1.2)
Education	
No formal education	47 (13.9)
Degree	
Elementary	36 (10.6)
Middle school	97 (28.6)
High school degree	135 (39.8)
College degree/Graduate	24 (7.1)
Marital status	
Unmarried	183 (54)
Married to	156 (46)
a man	26 (7.7)
a woman	125 (36.8)
both a man and a woman	5 (1.5)
Self categorized sexual orientation	
Bisexual	40 (11.8)
Ta	31 (9.1)
Straight men	30 (8.8)
Homosexual/gay	45(13.3)
Meti	193 (56.9)
Participation in an HIV prevention intervention in the past year	291 (85.8)

a Also reporting other occupation such as student or private company staff.

**Figure 3:**
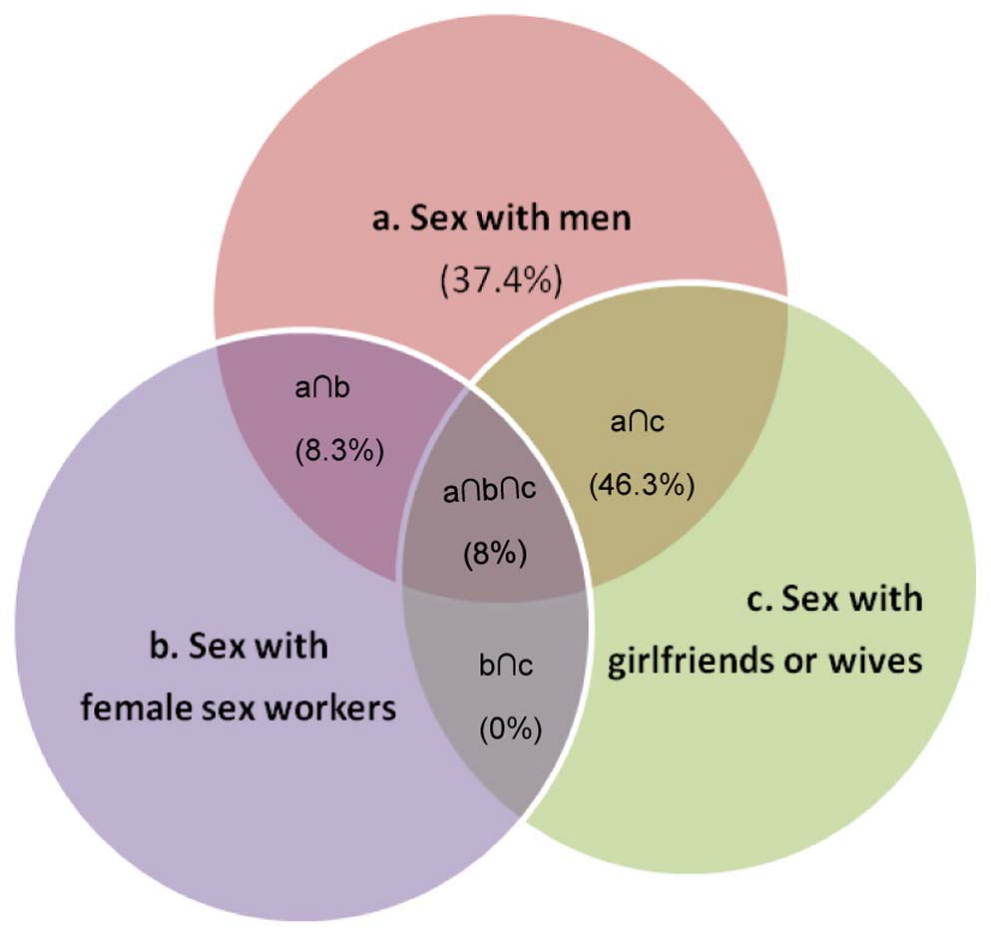
Venn diagram illustrating the overlap of sexual relationships (anal/oral sex with men, vaginal/oral/anal sex with female sex workers, girlfriends or wives) in the last 12 months among 339 surveyed MSM. ∩ represents an intersection between two groups (e.g. ‘a’ = sex with men only while, a∩b = sex with both men and female sex workers).

Figure 3 depicts the overlap of sexual relationships in last 12 months among the 339 MSM who were surveyed. Almost half (46%) of the total participants had had sex with both men and a female partner (wife or girlfriend) in past 12 months and 8% of the participants reported that they also had had sex with male, wives/girlfriends and female sex workers over the same period (Figure 3).

### Psychosocial health problems and other socio-behavioral factors

**Figure 4:**
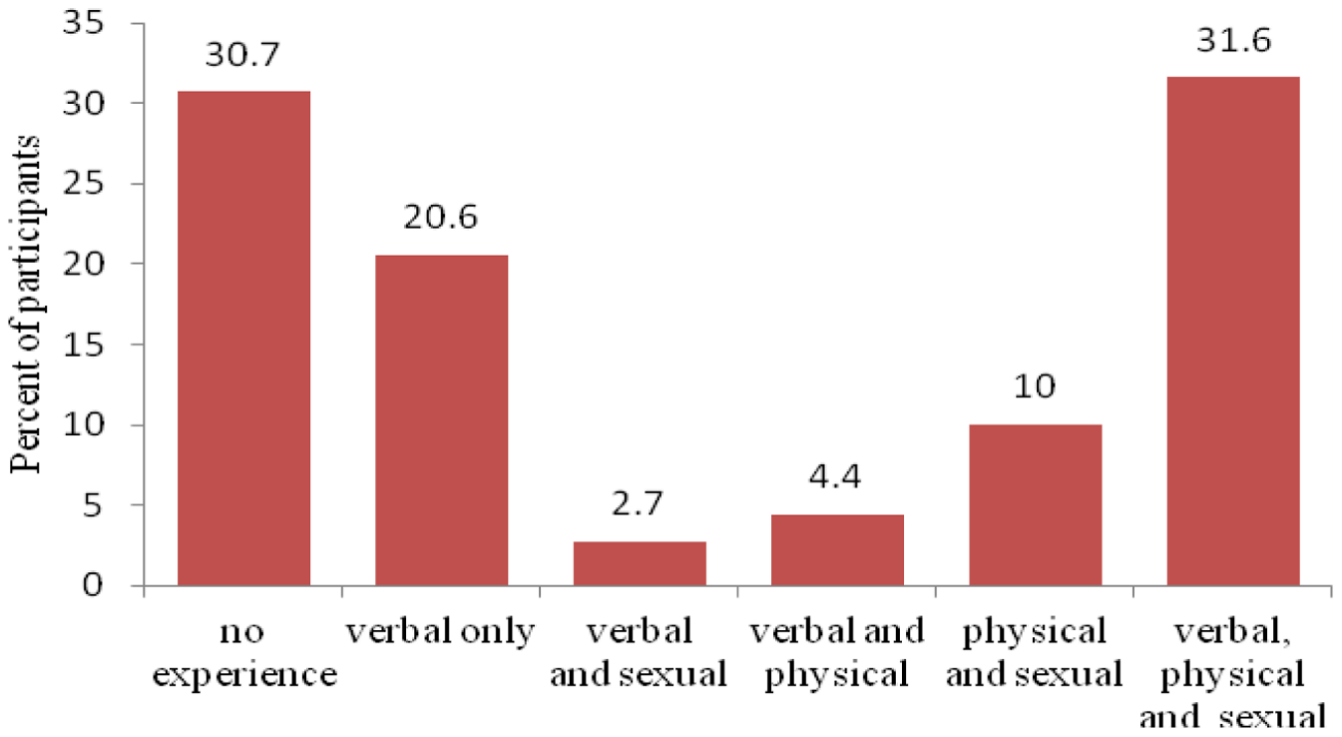
Experience of violence in the past year among 339 surveyed MSM in Nepal.

One fifth (21%) of the participants reported experiencing verbal abuse, 10% reported experiencing physical and sexual abuse, and 32% reported experiencing all three forms of abuse (Figure 4). When asked about the person responsible for the abuse, 38% reported physical abuse by a spouse/sexual partner/relative and 41% also reported sexual abuse or assault by a spouse/sexual partner/relative (Table 2).

**Table 2:** Sexual behavior, psychosocial health problems and other socio-behavioral factors among 339 MSM surveyed in Nepal.

Characteristic	N (%)
MSM who did not use condom for anal sex with at least one man in the last three sexual encounters	265 (78.2)
MSM who did not use condom with a woman in their last sexual encounter (N = 150)	118 (78.7)
Depression (CES-D)^a^	
Yes	206 (60.8)
Physical abuse by spouse/relative/sexual partners (Yes)	130 (38.3)
Sexual abuse by spouse/relatives/sexual partner (Yes)	138 (40.7)
Substance use disorders	
Alcohol abuse (yes)	75 (22.1)
Alcohol dependence (yes)	80 (23.6)
Drug abuse (yes)	5 (1.5)
Drug dependence (yes)	5 (1.5)
Social support scale	
SSQN^b^ mean/SD^c^ (range)	3.43/1.81 (0–9)
SSQS^d^ mean/SD (range)	4.61/1.06 (0–6)
Family acceptance	
Yes	146 (43.1)
No	193 (56.9)
Suicidality	
Suicidal ideation (yes)	159 (46.9)
Frequency of suicide ideation	
Always	79 (23.3)
Sometimes	57 (16.8)
1 or 2 times	23 (6.8)
Ever made a suicide plan (yes)	134 (39.5)

a CES-D: Center for Epidemiologic Studies Depression Scale.

b SSQN: social support questionnaire number score.

c SD: standard deviation.

d SSQS: social support questionnaire satisfaction score.

The prevalence of depression was 61% among the participants (Table 2). As we assessed abuse regardless of whether dependence was present and vice versa, the prevalence of alcohol dependence during the past year (24%) was slightly higher than that of the alcohol abuse (22%). A small percentage of the participants (2%) were abusing and dependent on illicit drugs (marijuana). The mean±SD of the SSQN and SSQS was 3.43±1.81, meaning study participants had between 3–4 supportive persons who could be counted on for different forms of social support and most were fairly satisfied with the available support 4.61±1.06. Four out of every 10 study participants reported that they had someone in their immediate family that they can talk openly about their homosexual/bisexual behavior. Nearly half of all participants (47%) reported ever having thought of committing suicide and 40% reported ever having made a suicide plan (Table 2).

### Variables associated with non-use of condoms

Overall, 78% of the participants reported non-use of condoms during anal sex in any of their last three sexual encounters with men, and a similarly high proportion also disregarded condoms when having anal and or vaginal sex with a woman in last encounter (Table 2).

Education (formal vs. informal) had a protective effect against non-use of condoms during anal sex with at least one man in the last three encounters, even after we controlled for marital status (married vs. single), age (continuous), religion (Hindu, Buddhist, Muslim, and Christian), and self-reported sexual orientation (adjusted odds ratio (AOR): 0.18, 95% CI:0.04–0.78, p = 0.022). Formal education was even more protective against non-use of condoms in the last encounter with a woman (AOR: 0.07, 95% CI: 0.01–0.69, p = 0.023) (data not shown). Non-participation in any HIV prevention intervention tripled the relative risk of unsafe anal sex (non-use of condoms) with at least one man in the last three encounters (AOR: 3.02, 95% CI: 1.11–8.20, p = 0.031) (Table 3). There was no significant increase in HIV risky behavior (non use of condoms) for those practicing both insertive and receptive anal roles (vs insertive only); nor for those practicing both receptive and oral roles, and for insertive, receptive and oral roles (data not shown). Nor did we find any significant association between unsafe sex behavior and alcohol abuse or alcohol dependence (Table 3).

**Table 3:** Non-use of condoms for anal sex by MSM in their last three sex encounters with men and associated factors.

Characteristic	Reported non-use of condom for anal sex with at least one man in the last three encounters	OR (95% CI)	AOR^a^ (95% CI)
	N (%), 265 (78.2)		
Education			
No formal education*	45 (16.9)	1	1
Formal education**	220 (83.1)	0.14 (0.03–0.57)	0.18 (0.04–0.78)†
Occupation			
Other*	136 (51.3)	1	1
Sex worker	129 (48.7)	1.18 (0.70–1.98)	0.81 (0.42–1.55)
Participation in any HIV prevention interventions			
Yes*	222 (83.8)	1	1
No	43 (16.2)	2.67 (1.02–7.01)	3.02 (1.11–8.20)†
Experience of violence			
No*	76 (28.7)	1	1
Verbal only	60 (22.7)	2.21 (0.99–4.91)	2.16 (0.75–6.23)
Verbal and physical	10 (3.8)	0.74 (0.23–2.35)	0.76 (0.19–2.98)
Physical and sexual	31 (11.7)	3.81 (1.08–13.45)	4.54 (1.02–20.18)†
Depression (CES-D)			
No*	95 (35.8)	1	1
Yes	170 (64.2)	1.89 (1.12–3.18)	1.89 (1.06–3.36)†
Alcohol abuse			
No*	27 (10.2)	1	1
Yes	57 (21.5)	1.07 (0.46–2.46)	1.11 (0.46–2.64)
Alcohol dependence			
No*	25 (9.4)	1	1
Yes	58 (21.9)	0.63 (0.23–1.75)	0.73 (0.24–2.24)
Social support scale			
SSQNb,c			
Q5^d^(high)*	58 (21.9)	1	1
Q4	52 (19.6)	1.12 (0.52–2.32)	1.06 (0.49–2.28)
Q3	68 (25.7)	1.45 (0.69–3.00)	1.52 (0.72–3.21)
Q2	46 (17.4)	1.11 (0.52–2.39)	1.05 (0.47–2.34)
Q1 (low)	41 (15.5)	3.71 (1.19–11.62)	3.49 (1.10–11.14)†
SSQSe,f			
≥5*	60 (22.6)	1	1
<5	205 (77.4)	2.42 (1.09–5.31)	2.30 (1.04–5.12)†
Suicidal ideation			
No*	141 (53.2)	1	1
Yes	124 (46.8)	0.98 (0.59–1.64)	0.83 (0.46–1.49)
Frequency of suicidal ideation			
Once or twice*	13 (4.9)	1	1
Sometimes	48 (18.1)	4.1 (1.38–12.19)	4.92 (1.48–16.29)†
Always	63 (23.8)	3.03 (1.13–8.15)	3.91 (1.29–11.87)†
Ever made a suicide plan			
No*	159 (60)	1	1
Yes	106 (40)	1.09 (0.65–1.86)	0.96 (0.53–1.72)

* Reference category

** Formal education includes elementary, middle school, high school degree, and college degree/graduate.

† p value less than 0.05

OR: Unadjusted Odds Ratio.

AOR: Adjusted Odds Ratio.

CI: Confidence interval.

CES-D: Center for Epidemiological Studies Depression Scale.

a Adjusted for age, religion, marital status and MSM subpopulation (*ta*, *meti*, bisexual, gay).

b SSQN: social support questionnaire number score, indicating number of supportive persons who can be counted on for different forms of social support.

c Skewness = 0.625 and kurtosis = 0.541.

d Cut-off point of quintile of SSQN Q1<2, Q2 (2–3), Q3 (3–4), Q4 (4–5) and Q>5.

e SSQS: social support questionnaire satisfaction score (1 =  very dissatisfied), (2 =  fairly dissatisfied), (3 =  a little dissatisfied), (4 =  a little satisfied), (5 =  fairly satisfied).

f Cut-off point based on sample median (Skewness = −2.63, Kurtosis = 7.58).

However, experience of physical and sexual violence (AOR: 4.54, 95% CI: 1.02–20.2, p = 0.047), depression (AOR:1.89, 95% CI:1.06–3.36, p = 0.031), sometimes having suicidal thoughts (AOR: 4.92, 95% CI: 1.48–16.29, p = 0.009), always having suicidal thoughts (AOR:3.91, 95% CI: 1.29–11.87, p = 0.016), poor social support network (0–2 persons) (AOR:3.49, 95% CI: 1.10–11.14, p = 0.035) and dissatisfaction with available social support (AOR:2.30, 95% CI:1.04–5.12, p = 0.041) were associated with non-use of condom for anal sex with at least one man in last three encounters (Table 3). Depression was marginally associated with non-use of condoms with women in last sexual encounter (AOR: 2.58, 95% CI: 0.98–6.79, p = 0.055) even after controlling for age, marital status, religion, and self-reported sexual orientation (data not shown).

MSM who reported non-use of condoms for anal sex with at least one man in last three encounters were significantly less likely to have participated in at least one HIV prevention intervention in the past year (AOR: 0.35, 95% CI: 0.13–0.93, p = 0.036). MSM who self-categorized their sexual orientation as *ta* and reported no family acceptance were also less likely to have participated in HIV prevention intervention (AOR: 0.29, 95% CI: 0.12–0.74, p = 0.009) vs. (AOR: 0.44, 95% CI: 0.21–0.93, p = 0,032) respectively (variables adjusted for each other) (Table 4).

**Table 4:** Factors associated with participation in any HIV prevention intervention in the past year among 339 MSM surveyed in Nepal.

Factors	OR (95% CI)	p-value	AOR^a^ (95% CI)	p-value
Reported non-use of condom for anal sex with at least one man in last three encounters				
No*	1		1	
Yes	0.37 (0.14–0.98)	0.046	0.35 (0.13–0.93)	0.036
Reported non-use of condom with woman in last encounter				
No*	1			
Yes	0.39 (0.11–1.37)	0.140		
Age^b^				
>25*	1			
≤25	0.94 (0.51–1.73)	0.840		
Education				
Formal education^c^	1			
No formal education	0.65 (0.29–1.45)	0.293		
Self-categorized sexual orientation				
Meti*	1		1	
Ta	0.24 (0.10–0.59)	0.002	0.29 (0.12–0.74)	0.009
Bisexual	0.46 (0.19–1.14)	0.094	0.55 (0.22–1.40)	0.211
Straight	0.38 (0.15–0.99)	0.049	0.44 (0.16–1.20)	0.110
Homosexual/gay	1.62 (0.46–5.70)	0.454	1.63 (0.46–5.84)	0.452
Ocupation				
Other*	1			
Sex worker	1.81 (0.96–3.42)	0.067		
Family acceptance of MSM identity				
Yes*	1			
No	0.34 (0.17–0.70)	0.003	0.44 (0.21–0.93)	0.032

* Reference category

OR: Unadjusted Odds Ratio.

AOR: Adjusted Odds Ratio.

CI: Confidence interval

a Final multivariable model includes all significant bivariate variables

b Dichotomization based on sample median

c Formal education includes elementary, middle school, high school degree, and college degree/graduate.

## Discussion

This study is the first to explore the role of psychosocial health problems and other socio-behavioral factors (social support and family acceptance) for HIV risk behavior among MSM in Nepal. The results indicate that high-risk behavior is very prevalent and that unsafe concurrent sexual relationships with both men and women are very common. We found that among MSM, experience of physical and sexual abuse, perceived low social support, depression and higher frequency of suicidal ideation were associated with increased non-use of condoms with men. On the other hand, formal education and participation in any HIV prevention interventions in the past year appeared strongly protective.

Over a third of surveyed MSM were married to women. The reasons for Nepalese MSM to have bisexual relationships have been explored previously [10]. Marriages between men are informal in Nepal, meaning that a marriage license is not obtained. However, the status of same sex marriage in Nepal is in a state of flux as the Supreme Court of Nepal decided in 2008 to grant all rights to MSM [28]. Alarmingly, most of the respondents reported experience of violence (verbal, physical and sexual) in the last 12 months and nearly 4 out of every 10 said they had experienced physical and sexual abuse from a spouse/relative or sexual partner. A previous study among Nepalese MSM carried out by the study team found that *metis* (most of them involved in sex work) are more likely to experience violence than other sex role MSM (*ta* and gay etc) due to their feminine nature, which renders them more visible in society [9]. A very high proportion, six out 10, felt depressed, which in itself was an independent predictor of unsafe anal sex. These findings add to emergent evidence suggesting a clear link between depression, experience or fear of violence and unsafe anal sex in MSM due to a compromised ability to negotiate condom use with sex partners [14], [15], [29]–[32]. Nearly half of all participants reported that they had thought of committing suicide. Similarly high proportions of the study participants also abused or were addicted to alcohol. Other studies, mostly from high-income countries, have also established that the combination of discrimination, violence and rejection is common among MSM and fuels their risk of substance disorders and mental health problems [33]–[36].

Related to family acceptance, men with little social support (scoring in the lower quintile of SSQN) and those dissatisfied with available support (SSQS median<5) were more likely to practice unsafe sex, in line with previous research showing that perceived high and satisfactory social support promotes mental well-being, less substance abuse [37], [38] and positive health behavior among MSM [39]. Most previous studies that analyzed associations between low social support or dissatisfaction with social support and unprotected anal sex in MSM were performed in western countries [40]–[42]. To our knowledge, this is the first study from a low-income setting of Asia to explore such associations among MSM. Our results also add to the growing bulk of evidence suggesting that with each additional psychosocial health problem, the odds of increased HIV risk behaviors also increases among MSM [43].

The syndemic we have described (substance use disorder, violence, depression, suicidality and low social support) not only increased HIV risk behavior, they also undermined the utilization of traditional HIV prevention interventions among hidden MSM [16], [44]. Our study found that MSM with risky sexual behavior, lacking family acceptance, and identifying as *tas* (i.e. the insertive role MSM) were less likely to have participated in an HIV prevention intervention. Experience of physical abuse among Nepalese MSM NGO outreach workers by police is an identified barrier for HIV prevention and care services [44]. Studies conducted among African MSM also found associations between safer sexual behavior and previous participation in HIV prevention and care services [45]. Similar to our findings, *Panthis* from India (corresponding to *tas* in Nepal) are also less likely to participate in HIV prevention programs because they are less likely to have accepted their MSM identity [46]. Our previous study among Nepalese MSM found that the strong social stigma against male-male sex forces many MSM (especially those who have an insertive sex role) to self-identify as heterosexuals and remain hidden [9]. This may be the reason why despite Nepal's extensive National HIV surveillance system and health outreach center efforts to reduce risky sexual behavior, Nepalese MSM continue to practice high HIV risk behavior [9]–[11]. Recent studies and reviews [47]–[50] have found that providing counseling to reduce stress or building stress management skills, and social support through peer groups, partner or family support, are effective at reducing HIV-related risk behaviors (e.g. non-use of condoms and high number of sexual partners) among MSM. Such interventions, in particular enhanced family/social support, could likely also be beneficial for Nepalese MSM. Increasing access to care and prevention in general through a strengthened health system, clear policy formulation and effective implementation and the protection of human rights for vulnerable populations are other key interventions. Finally, understanding determinants of self-stigma and enacted stigma among Nepalese MSM will help in designing programs that address this group's psychosocial health needs more comprehensively. 

Three limitations of our study need to be taken into account when interpreting the study results. First, the cross-sectional study design undermines the ability to draw conclusions about causality. Second, the convenience sampling restricts the generalizability of the study findings. The MSM recruited were those accessible through NGO outreach workers, indicating that this sample could have been the relatively more visible MSM or MSM who are more aware of HIV prevention interventions. Thus, they may not be representative of the entire Nepalese MSM community and our findings may therefore underestimate risk behaviors in even more hidden MSM. Third, using an interviewer-administered questionnaire increases the risk of social desirability, and in turn, possibly underestimates actual risk behavior among MSM.

## Conclusions

In summary, among Nepalese MSM, unprotected sex with both men and women was very common and strongly associated with psychosocial health problems and other psychosocial factors. Higher education and participation in HIV prevention interventions protected against non-condom use. However, those most vulnerable with the highest risk behavior are not reached by current prevention efforts or choose to not take up safer sexual behaviors. Integrating and addressing psychosocial health problems and other socio-behavioral factors in the national response to HIV prevention could enhance the effectiveness of existing behavioral HIV prevention interventions targeting MSM in Nepal.

## Supporting Information

Appendix S1 **Oral informed consent.**
(DOCX)
