# Supplementary material for: Psychosocial Health Problems Associated with Increased HIV Risk Behavior among Men Who Have Sex with Men in Nepal: A Cross-Sectional Survey
Source: PLoS One. 2013 Mar 13;8(3):e58099. doi: 10.1371/journal.pone.0058099 (PMC3596342; doi:10.1371/journal.pone.0058099)
Supplement: Appendix S1 — Oral informed consent. (DOCX) [file pone.0058099.s001.docx]

**Introduction**

Hi, My name is ................. You are being asked to take part in this research study to collect information on HIV risk behaviors and psychosocial health problems among the populations like you. This study is funded by the Netherlands Development Organization (SNV) through Social Inclusion Research Fund. This is a consent form. It gives you information about this study. I will explain the purpose of the study and the kinds of activities that are part of this study. I will describe the risks and benefits of the study. I will describe what is expected of you, if you decide to participate. This consent form might contain some words that you do not know. Please ask me to explain anything that you do not understand. After I tell you about the study I will ask if you want to be part of the study. If you agree to take part, then I will start interview.

Please note that:

- Your participation in this research is entirely voluntary.
- You may decide not to take part or to withdraw from the study at any time.

**Purpose of the study**

The main purpose of the study is to assess psychosocial health problems associated with increased HIV risk behaviours among men who have Sex with Men (MSM) in Nepal. To be specific, the study assess whether there is relationship between psychosocial health problems and HIV risk behaviors among MSM.

**Risks and discomfort**

You will be asked questions about your sexual history. You may be embarrassed by these questions. You may choose not to answer the questions, if you wish. I will make every effort to protect your confidentiality during the study. However, it is possible that others might learn of your participation and think you are infected with HIV or are at high risk for HIV infection. Because of this, you could have problems being accepted in your family and community.

**Potential benefits**

There may be no direct benefit for you as a result of participating in this study. However, what we learn from this study may help scientific community and program planners to find new ways to prevent the spread of HIV in the future. Results of this study may help to answer the question- Is there relationship between psychosocial health problems and HIV risk behaviours among MSM. You will receive information about how to protect yourself and your partner(s) from HIV and other sexually transmitted infections (STIs). You will also receive information about the places (public or non-governmental organizations health institutions or care and support centres) to get the appropriate treatment free of cost for self-reported STIs or possible psychosocial health problems.

**Leaving the study**

You have a right to leave the research at any time without any penalty or future disadvantage whatsoever.

**Study findings**

At the end of the interview, you will be told when the study results may be available and how to learn about them.

**Reimbursement**

I will not pay you for your participation in the study.

**Confidentiality**

I will do everything to keep your personal information confidential. Any publication that results from this study will not use your name or identify you personally. I will not ask you about your any personal identifiers such as name, living address or contact address etc. Some people may review each questionnaire to ensure that this research is done properly; these include: SNV, Nepal and research project supervisor assigned by the SNV, Nepal. .

Would you be willing to participate?

1. Yes 2. No

For any other queries, please contact: Principal investigator: Keshab Deuba; Email: [deuba4k@gmail.com](mailto:deuba4k@gmail.com); Mobile: 9841259990.
